# Vaccines against Infectious Diseases and Cancer

**DOI:** 10.3390/vaccines10050648

**Published:** 2022-04-20

**Authors:** Ângela Sousa, Christiane P. Soares, Aldo Venuti

**Affiliations:** 1 CICS-UBI—Health Sciences Research Centre, Universidade da Beira Interior, 6201-506 Covilhã, Portugal; 2 Department of Clinical Analysis, School of Pharmaceutical Sciences, São Paulo State University (UNESP), Rodovia Araraquara/Jaú Km 1, No Number, Campus Ville, Araraquara 14800-903, São Paulo, Brazil; 3 HPV-UNIT-UOSD Tumor Immunology and Immunotherapy, IRCCS Regina Elena National Cancer Institute, 00144 Rome, Italy

We live on a planet marked by remarkable health disparities. Population migration brings those disparities into contact, increasing infectious and communicable disease epidemiology. In addition, the action mechanism of some infectious agents (such as bacteria, viruses, and parasites) can convert an infected cell into a cancer cell. Consequently, infectious diseases are becoming a huge public health problem, not only due to the impact that a new worldwide outbreak can bring to heath systems and global economy but also due to the risk of progression into cancer diseases.

The development of effective and specific vaccines against each infectious agent requires a broad understanding of pathogens’ molecular targets and hosts’ immune response towards that pathogen in order to induce the activation and cooperation of multiple components of both the innate and adaptive immune systems. In particular, vaccine formulations able to efficiently stimulate T and B cells are fundamental to adaptive immune responses and offer preventive and therapeutic immunity.

The Special Issue entitled “Vaccines against Infectious Diseases and Cancer” contains articles, reviews, brief reports and a meta-analysis to give a perspective on recent advances in the design of efficient vaccines against emergent infectious pathogens, focusing on the most adequate vaccine typology (viral or non-viral), manufacturing technologies, computational modelling approaches, delivery systems, adjuvants, administration routes and the respective induced immune responses.

Pegivirus is an enveloped, positive-stranded RNA and lymphotropic virus, leading up to 25% of persistent infections in worldwide, since no effective therapeutic means are available to date. Zheng et al. designed a vaccine ensemble by mapping the whole proteome-based mining of immunogenic peptides, from CTL (cytotoxic T lymphocytes), HTL (helper T lymphocytes), and B cell epitopes, using computational modelling approaches [1]. They identified 29 different epitopes with a critical role in immune response induction and confirmed the induction of both primary and secondary immune factors such as IL, cytokines and antibodies and by in silico expression and host immune simulation.

Melioidosis is induced by the *Burkholderia pseudomallei* (*B. pseudomallei*) infectious bacterium, reaching up to a 50% mortality rate due to the lack of an available vaccine. Grund et al. explored bioinformatic tools to predict potential epitopes from an outer membrane protein of the *B. pseudomallei* (Bucl8), ensuring that they are non-allergenic and non-toxic, but elicit an antigen-specific immune response [2]. They formulated a vaccine based on two extracellular components of Bucl8, the β-barrel loops and extended collagen and non-collagen domains. After the mice vaccination, they observed that the immunization with synthetic loop peptides had a stronger, more consistent antibody response than recombinant protein antigens.

A health emergency with major social and economic disruptions has spread worldwide since December 2019, when the first cases of COVID-19 disease induced by severe acute respiratory syndrome coronavirus (SARS-CoV-2) appeared. The first approved vaccine against COVID-19 is based on the messenger RNA-1273 to express S protein (BNT162b2 mRNA COVID-19 Vaccine) and showed for the first time the efficacy, safety and rapid manufacturing of nucleic acid vaccines instead of viral vaccines [3]. After this approval, a vaccine mass production began in several countries of different continents, to speed up the COVID-19 vaccination program in all countries of the world. However, a recent study showed a strong linear association between per capita income and the proportion of people vaccinated in countries with populations of one million or more [4]. For instance, rich European and North American countries are most protected against COVID-19, while there is a significant disparity among Asian countries, and less developed African countries barely initiated a vaccination program. 

Other particular studies were based on the reactogenicity and SARS-CoV-2 antibodies induction of the BNT162b2 mRNA COVID-19 Vaccine. Pellini et al. verified that 21 days after the first dose, the BNT162b2 vaccine activated immune responses in 98% of the participants and the antibody titer was greater in younger (<38 years) vs. older participants (<38 vs. 47–56 *p* = 0.002; <38 vs. >56 *p* = 0.001) [5]. Rahmani et al. investigated the adverse reactions occurring in the 7 days following the first and second vaccination doses, and they also concluded greater reactogenicity among the young age population, with a higher prevalence in females [6]. Campo et al. studied the immunogenicity data 6 months after the first dose of BNT162b2 in correlation with age, gender, body mass index, comorbidities and previous SARS-CoV-2 infection [7]. In general, they observed that some variables seemed to influence antibody levels, in particular the age and previous COVID-19 infection. In addition, they clearly showed antibody persistence at 6 months, albeit with a certain decline, which in turn opens a debate about the need for further boosts. 

Given that patients with cancer are among the most vulnerable groups of the COVID-19 pandemic, a systematic review and meta-analysis was performed to assess the seroconversion rate and the safety of COVID-19 vaccinations in cancer patients until 31 July 2021 [8]. The study indicated that vaccines had a good safety profile, and although the antibody titers of patients with solid tumors were significantly lower than those of healthy controls, they revealed adequate antibody responses (>90%). In patients with hematologic malignances, a significantly lower rate of seroconversion was registered. Furthermore, the COVID-19 pandemic has strongly impacted on public health services, particularly on vaccinations that, especially during the first pandemic phase, have been often delayed and/or canceled. The most affected vaccinations by the pandemic have been the non-mandatory ones, particularly those addressing the adolescent and adult population, such as prophylactic immunization against papillomavirus (HPV) [9]. In this way, several strategies have been implemented to increase overall vaccination coverage rates and avoid the persistent infection by HPV, which, consequently, can result in benign pathologies or cancers both in males and females. The carcinogenic process induced by this virus mainly depends on the overexpression of the HPV E6 and E7 oncoproteins, which interfere with cell cycle regulation and proliferation through the impairment of tumor suppressor proteins, such as p53 and pRb, respectively [10,11]. Given the available prophylactic vaccines based on virus-like particles do not exert any therapeutic effect against an ongoing HPV infection, there is a great need to explore suitable alternatives to treat cancers induced by this virus, such as anti-HPV drugs based on small natural or synthetic molecules and therapeutic DNA vaccines [12,13]. However, no paper within this special issue was dealing with therapeutic vaccines against virus-associated cancer, most probably because the SARSCoV2 pandemic moved many research groups to work on infectious diseases. 

On the other hand, the pandemic situation induced by COVID-19 has stressed the relevance of vaccination programs and the urgency of working on new technologies that allow an efficient, safe, and effective immunization. For instance, Lundstrom wrote a review focused on some innovation in viral vaccines typology, especially the self-replicating RNA viruses and their advantages in vaccine development against infectious diseases and cancer [14]. In this case, the subgenomic RNA, due to the presence of RNA-dependent RNA polymerase activity, can replicate close to 10^6^ copies per cell for translation in the cytoplasm providing extreme transgene expression levels. This property allows the administration of these vaccines as replicon RNAs at significantly lower doses than conventional mRNA, recombinant particles or DNA plasmids, showing safety and robust immune response and protection in animal models.

Most licensed vaccines have intrinsic limitations, such as variable efficacy, adverse effects and some shortcomings, emphasizing the need for exploring potential solutions. Emerging vaccine technologies involving nanoparticles such as self-assembling proteins, virus-like particles, liposomes, virosomes and polymeric nanoparticles offer novel, safe and high-potential approaches to address many vaccine-development-related challenges. Considering that nanomaterials provide effective vehicles for antigen delivery and immunostimulatory agents to accelerate the vaccine evolution, Celis-Giraldo et al. discussed the requirements for an effective, broad-coverage-elicited immune response, the main nanoplatforms for producing it and the latest nanovaccine applications for fighting animal pathogens [15].

Among the challenges of new vaccine generations is the search for alternative routes of antigen delivery due to costs, risks, the need for trained personnel and low acceptance in the population associated with the parenteral route. Valdivia-Olivares et al. pointed out that transdermal immunization has been suggested as a promising alternative for antigen delivery and vaccination based on a large absorption surface and an abundance of immune system cells, which contribute to a high barrier capacity and high immunological efficiency [16]. They also highlighted the antigen passage facilitated by devices and the importance of nanosystems’ design and composition towards the new generation of needle-free nanometric transdermal systems. 

In addition, the innovative technology of Cre-recombinase-mediated in vivo minicircle DNA vaccination (CRIM) offers an advantageous option to replace a traditional DNA vaccine. Wang et al. designed a dual promoter expression plasmid, which can synthesize a short antigen protein under a prokaryotic in vivo promoter and full length antigen protein under the eukaryotic promoter at the same time [17]. Using the self-lysed Salmonella strain as a delivery vesicle, they provided 80% protection to chickens immunized with the CRIM vector with a dual promoter when compared to the 50% protection as result of the CRIM vector containing only the eukaryotic promoter.

Altogether, the articles, reviews, brief reports, meta-analysis and perspective within this Special Issue on “Vaccines against Infectious Diseases and Cancer” describe the impact that emergent pathogens, such as SARS-CoV-2, induced in the global evolution of vaccines, in socio-economic disruption and in public health services. In addition, this Special Issue also highlights advances in the antigen design resorting to bioinformatics and different vaccine typologies, manufacturing technologies, delivery systems, adjuvants and administration routes that create the potential for new vaccine platforms to overcome licensed vaccines-related challenges.

## Data Availability

The data presented in this study are available on request from the corresponding author.
